# Host density and habitat structure influence host contact rates and *Batrachochytrium salamandrivorans* transmission

**DOI:** 10.1038/s41598-020-62351-x

**Published:** 2020-03-27

**Authors:** Daniel A. Malagon, Luis A. Melara, Olivia F. Prosper, Suzanne Lenhart, Edward Davis Carter, J. A. Fordyce, Anna C. Peterson, Debra L. Miller, Matthew J. Gray

**Affiliations:** 1 Center for Wildlife Health, Department of Forestry, Wildlife and Fisheries, University of Tennessee Institute of Agriculture, Knoxville, TN 37996 USA; 2 Department of Mathematics, Shippensburg University, Shippensburg, PA 17257 USA; 3 Department of Mathematics, University of Kentucky, Lexington, KY 40506 USA; 4 Department of Mathematics, University of Tennessee, Knoxville, TN 37996 USA; 5 Department of Ecology and Evolutionary Biology, University of Tennessee, Knoxville, TN 37996 USA; 6 Department of Biomedical and Diagnostic Sciences, College of Veterinary Medicine, University of Tennessee, Knoxville, TN 37996 USA

**Keywords:** Freshwater ecology, Conservation biology

## Abstract

*Batrachochytrium salamandrivorans* (*Bsal*) is an emerging invasive pathogen that is highly pathogenic to salamander species. Modeling infection dynamics in this system can facilitate proactive efforts to mitigate this pathogen's impact on North American species. Given its widespread distribution and high abundance, the eastern newt (*Notophthalmus viridescens*) has the potential to significantly influence *Bsal* epidemiology. We designed experiments to 1) estimate contact rates given different host densities and habitat structure and 2) estimate the probability of transmission from infected to susceptible individuals. Using parameter estimates from data generated during these experiments, we modeled infection and disease outcomes for a population of newts using a system of differential equations. We found that host contact rates were density-dependent, and that adding habitat structure reduced contacts. The probability of *Bsal* transmission given contact between newts was very high (>90%) even at early stages of infection. Our simulations show rapid transmission of *Bsal* among individuals following pathogen introduction, with infection prevalence exceeding 90% within one month and >80% mortality of newts in three months. Estimates of basic reproductive rate (R_0_) of *Bsal* for eastern newts were 1.9 and 3.2 for complex and simple habitats, respectively. Although reducing host density and increasing habitat complexity might decrease transmission, these management strategies may be ineffective at stopping *Bsal* invasion in eastern newt populations due to this species’ hyper-susceptibility.

## Introduction

Across a variety of taxa, disease has been implicated as a major contributor to population- and species-level declines^1–6^. Epidemiological modeling can facilitate disease response and management by elucidating host-pathogen interactions and identifying strategies that could reduce the severity of outbreaks in wild populations^7–9^. Ideally, evaluating disease management strategies and modeling possible outcomes should occur prior to pathogen invasion, because the likelihood for disease control is greater and the cost of response is less^8,10–12^. Conversely, reactive or delayed responses to disease outbreaks can result in significant biodiversity loss and economic impact, as demonstrated by the unexpected emergence of *Batrachochytrium dendrobatids* (*Bd*)^6,13,14^ and *Pseudogymnoascus destructans* (the causative agent of White Nose syndrome)^13,15^.

The newly emergent fungal pathogen *Batrachochytrium salamandrivorans* (*Bsal*) provides a unique opportunity to evaluate possible management strategies, especially in areas where it has yet to emerge. *Bsal* is rapidly spreading in Europe, where it is believed to have been introduced from Asia via the pet trade^16,17^. In areas where *Bsal* has emerged, populations of fire salamanders (*Salamandra salamandra*) have declined substantially^18^. Preventing and mitigating *Bsal* outbreaks is described as one of the greatest current priorities for wildlife conservation^19^. *Bsal* appears to have a high invasion probability and has already been detected in wild populations of salamanders in several European countries^16,20,21^, within captive populations in western Europe^22,23^, and in the pet trade^24,25^. Currently, *Bsal* has not yet been detected in North America, though several risk assessment models predict *Bsal* invasion probability is high due to suitable environmental conditions, high salamander diversity, and its high likelihood of entry through trade^26–28^.

Initial model simulations using European fire salamanders predict *Bsal* outbreaks at low host densities and rapid spread of the pathogen across a landscape, suggesting that mitigation efforts should focus on preventing pathogen introduction and transmission within populations^29^. Although these simulations are useful for informing disease response options for the fire salamander, they may not be translatable to North American ecosystems, where susceptible species have different life history strategies^30–32^. North America is home to the greatest biodiversity of salamanders in the world^28^. In eastern North America, one of the most widely distributed and common salamander species is the eastern newt (*Notophthalmus viridescens*^31,32^). The eastern newt is as susceptible to *Bsal* infection as the fire salamander^16,33^; hence, this species could play a major role in the epidemiology of *Bsal* if the pathogen is introduced to North America.

In this study, we develop an epidemiological model to understand *Bsal* transmission and mortality within a population of eastern newts. We parameterize this model using host contact rates and *Bsal* transmission probabilities estimated during two laboratory experiments. In the first experiment, we estimated contact rates of newts among different host densities and levels of habitat complexity, as prior work has demonstrated that both of these factors can influence host contacts and within-system pathogen transmission^34–40^. We hypothesized that contact rates would increase linearly with host density (i.e., mass-action density dependence), as seen in many wildlife disease systems^36,41^. Theory predicts that mass-action density dependence can lead to a stable equilibrium, with hosts persisting at low densities^42^. We also hypothesized that increasing habitat complexity would reduce host contacts by providing greater opportunity for spatial separation^40^. In the second experiment, we evaluated whether the probability of *Bsal* transmission from an infected to susceptible host via contact changed among different durations of disease progression (i.e., disease states) and among different rates of direct contact between susceptible and infected hosts. We hypothesized that hosts infected with *Bsal* for a greater amount of time would cause greater rates of transmission, because pathogen loads should be greater as disease progresses^18,43–45^. We also hypothesized that as host contact rate increased, pathogen transmission would increase^46^. Using contact and transmission estimates from these experiments, we simulated infection prevalence and mortality in an eastern newt population over three months, and estimated the basic reproductive rate (R_0_) for *Bsal* in simple and complex habitats^42^.

## Results

**Figure 1:**
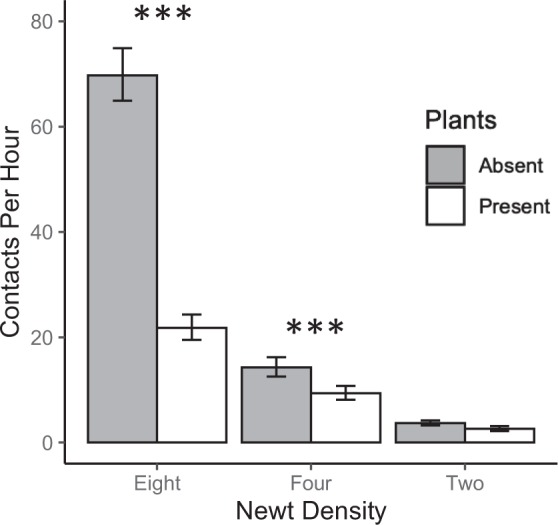
Estimated number of contacts per hour among three eastern newt densities per m^2^ when plants are absent (shaded bars) or present (9 plants/m^2^; white bars). Asterisks denote significant differences in host contact rates between habitat complexity treatments. Figure was produced from raw data with standard error bars based on back-transformed data used to construct linear models.

**Figure 2:**
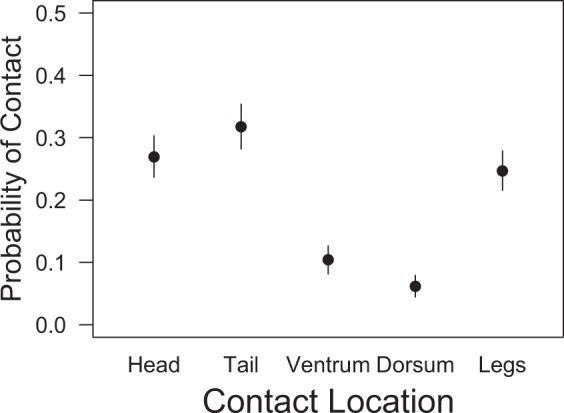
Estimates of the proportion of contacts between eastern newts among major body regions. Dots indicate the median of the posterior probability and bars indicate the 95% highest density interval.

Number of contacts per hour between newts was density dependent regardless of whether plants were absent (F_2,81_ = 199.3, *P* < 0.001) or present (F_2,81_ = 59.8, *P* < 0.001) in the mesocosms (Fig. 1). Per capita contact rates at 8 newts per m^2^ were 5–15X greater than 2 newts per m^2^. At 8 newts per m^2^, the presence of plants significantly reduced newt contacts by 3X(F_1,58_ = 80.3, *P* < 0.001), and at 4 newts per m^2^, plants reduced newt contacts by 35% (F_1,52_ = 4.89, *P* = 0.03; Fig. 1). Head, tail and leg contacts between newts were more common than ventrum and dorsum contacts (Fig. 2).

**Figure 3:**
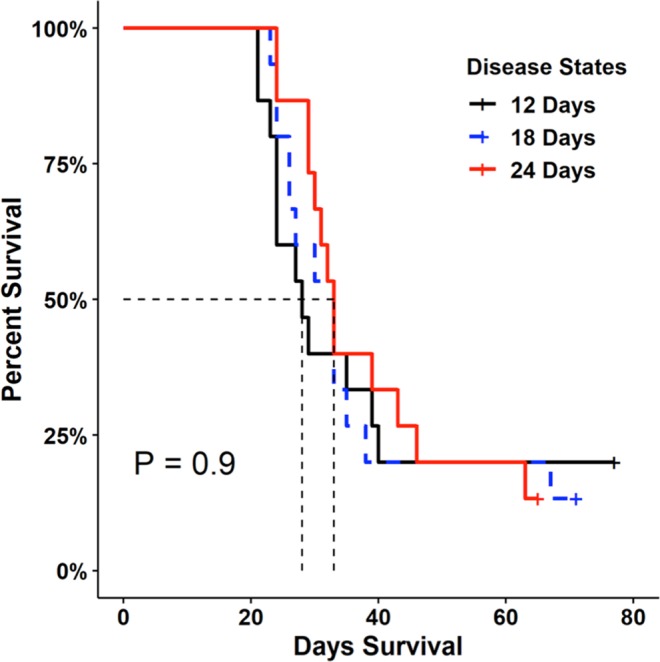
Survival curves for susceptible hosts following contact with an infected host among different infected host disease states (12, 18, or 24 days post-exposure to *Bsal*); dashed lines represent 50% mortality. Survival probabilities did not differ significantly (*P* = 0.90) among disease states using Kaplan-Meier analysis.

**Figure 4:**
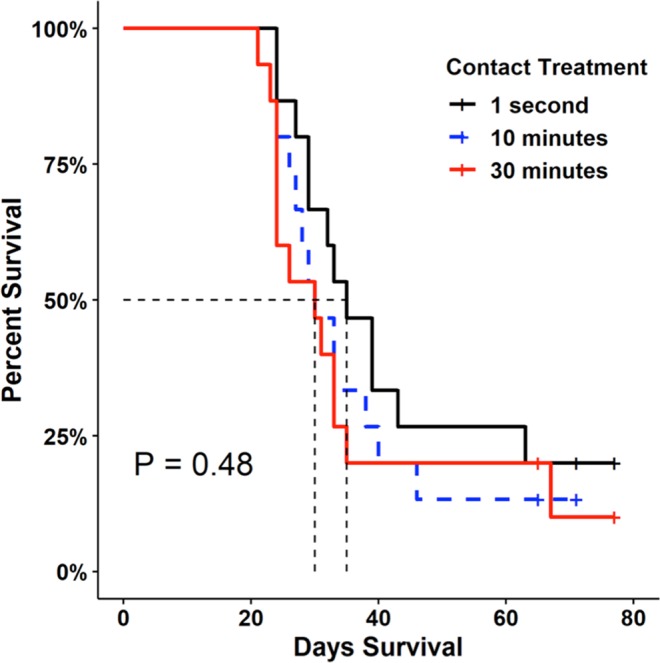
Survival curves of susceptible hosts following contact with an infected host among different contact rates (one-second forced contact, 10-min cohabitation, or 30-min cohabitation); dashed lines represent 50% mortality. Survival probabilities did not differ significantly (*P* = 0.48) among host contact treatments by Kaplan-Meier analysis.

**Figure 5:**
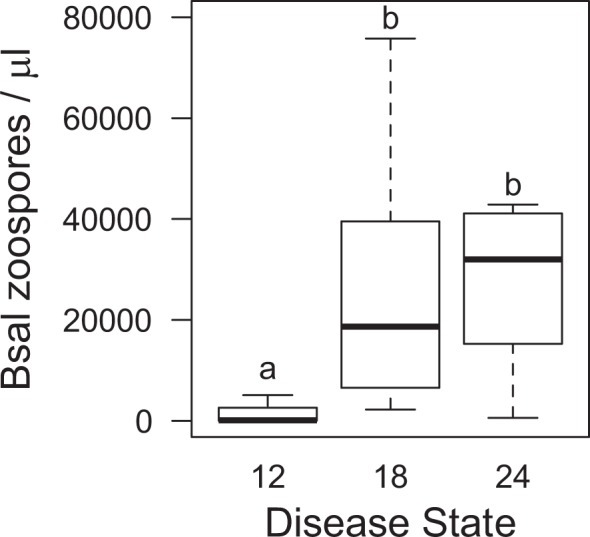
Infection loads of *Bsal* (copies/ uL) on the skin of infected newts that were exposed to susceptible newts at three states of disease progression (12, 18, or 24 days post-exposure to *Bsal*). *Bsal* loads in the infected hosts increased significantly with disease progression. Unlike letters above each disease state indicate significant differences detected in loads.

All susceptible newts (100%) became infected with *Bsal* following one or more contacts with an infected newt; the median duration to detectable infection was 7.5 days. Mortality of susceptible hosts began 22 days after contact with an infected host and increased rapidly thereafter (Figs. 3 and 4). Median duration to mortality was 32 days post-contact with an infected individual, with 89% cumulative mortality among contact treatments. Of the surviving newts (11%), all appeared to clear *Bsal* infection by the end of the 90-day experiment. Survival was similar among disease-state treatments (*X*^2^_2_ = 0.2, *P* = 0.92; Fig. 3), despite that infected hosts had greater *Bsal* loads on their skin at 24 days compared to 12 days of disease progression (F_2,7.8_ = 7.3, *P* = 0.02; Fig. 5). Survival of susceptible hosts also was similar among contact-rate treatments (*X*^2^_2_ = 1.4, *P* = 0.49; Fig. 4) – one contact for <1 second was sufficient to result in *Bsal* transmission from an infected to susceptible host.

**Figure 6:**
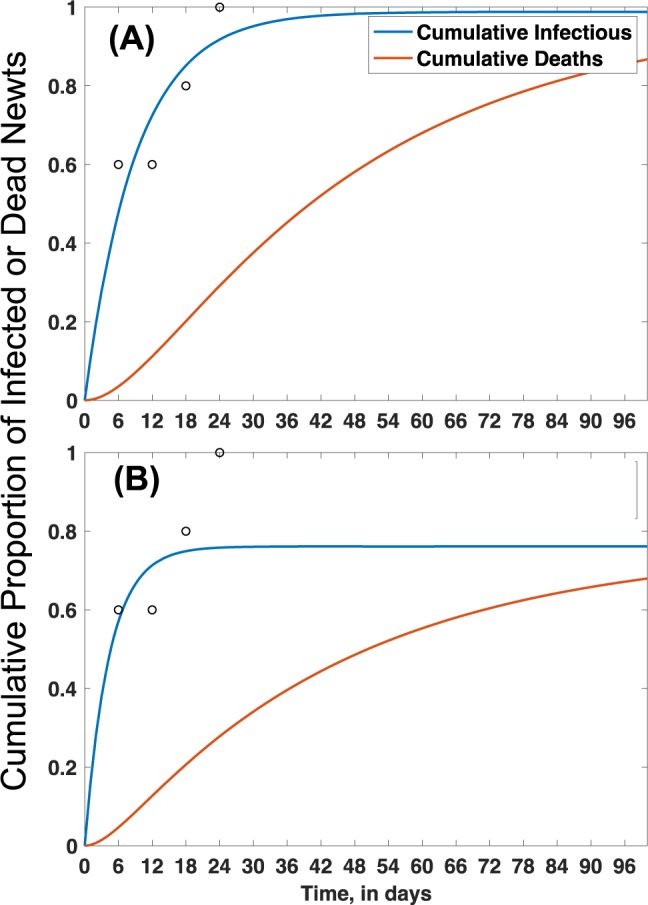
Cumulative proportion of infected and dead newts in a population simulated for 96 days following introduction of one infected adult eastern newt with *Bsal* transmission only occurring due to host-host contact. **(A)** Predicted dynamics in simple habitats (i.e., no plant treatment), and **(B)** complex habitats (i.e., 9 plants per m^2^) when contact is made with an infected host 12 days after initial exposure to *Bsal*. Open circles represent actual deaths recorded during experiments used to estimate model parameters (Supplementary Table S1).

Using data from the these experiments, the functional form (Holling’s Type II) of the density dependent contact rate (per hour) was found and used with a system of differential equations to model infection and disease dynamics in a population of eastern newts. In an aquatic habitat with simple complexity (i.e., no plants), our simulations suggest that >95% of newts could become infected after one month following introduction of one infected newt, with >80% mortality in three months (Fig. 6). In more complex aquatic habitat (i.e., 9 plants per m^2^), infection prevalence was <80% and cumulative mortality was <70% over the same duration (Fig. 6). Using the cumulative proportion of infected individuals in Fig. 6 (I_simple_ = 0.95, I_complex_ = 0.77), we estimated R_o_ as 1.9 and 3.2 for complex and simple habitats, respectively.

## Discussion

Our epidemiological model confirms that the eastern newt will likely play a major role in the epidemiology of *Bsal* if the pathogen emerges in North America^33^. Moreover, this abundant and widely distributed species could experience precipitous declines. The simulations show rapid transmission of *Bsal* among individuals, such that >90% of a population could become infected in less than one month, and mortality could exceed 80% in three months. Our results are similar to infection prevalence and mortality rates observed in wild fire salamander populations in Belgium^18^; however, the role of eastern newts in the epidemiology of *Bsal* in North America could be even more extreme. Our estimates of R_0_ exceeded one for eastern newts in all cases, which is similar to that estimated by Islam *et al*.^47^. The R_0_ is an estimate of secondary infections resulting from one infected salamander, hence R_0_ > 1 indicates infection will spread and an outbreak will occur^42^. As discussed below, the high contact rates and high probability of infection given contact certainly facilitate the likelihood of *Bsal* invasion in an eastern newt population. However, other aspects of eastern newt life history, might contribute to their role in *Bsal* epidemiology if the pathogen is introduced to North America. Eastern newts have a unique 3-stage life history where gregarious congregations of adults breed aquatically (hence facilitating density independent transmission), they lay eggs that develop into aquatic larvae, and larvae metamorphose to a juvenile eft stage that is terrestrial for up to eight years^48,49^. Eastern newt efts have high dispersal ability^49,50^, and they are susceptible to *Bsal* (MJG, unpubl. data), hence could play a role in the overland movement of *Bsal* among aquatic breeding sites and contamination of the terrestrial environment. Adult eastern newts can remain in the aquatic system or return to the terrestrial environment^49^, providing additional opportunities for maintenance and amplification of *Bsal* in both ecosystems. In comparison, fire salamanders are terrestrial species that interact with the aquatic environment only when viviparous larvae are born^30,51^, hence spillover and maintenance in the aquatic ecosystem is less likely than eastern newts. Despite potentially fewer transmission pathways than eastern newts, *Bsal* is devastating fire salamanders in Europe, which is not surprising because the estimated R_0_ = 9^45^. The difference in R_0_ between these species may be related to their susceptibility to *Bsal* infection. Although both species are very susceptible to *Bsal*, the infectious dose (ID)-50 for eastern newts is approximately 3000 zoospores (MJG, unpubl. data); whereas, fire salamanders can be infected with as few as 100 zoospores^18^. Given the clear and present threat of *Bsal* to eastern newts, we recommend that future planning for *Bsal* invasion and intervention in North America take into consideration the possible epidemiological role of this species in both aquatic and terrestrial ecosystems^33^.

We found that contact rates of adult eastern newts in an aquatic environment were density dependent, and that relationship was stronger when habitat structure was less complex. In fact, the addition of plants to the aquatic environment reduced newt contact rates by 3X at the highest newt density (8 individuals/m^2^) tested. Our simulations also suggest that complex habitats might reduce *Bsal* infection prevalence and prevent population extirpation. Greer *et al*. (2008) hypothesized that pathogen transmission in wetlands grazed by cattle was higher due to reduced habitat structure and increased contact rates^40^. Similarly, Becker and Zamudio (2010) found that *Bd* spread more slowly in fragmented landscapes where host contacts between patches occurred less frequently^52^. Indeed, complex habitats might provide barriers to movement and additional sites for refugia, resulting in decreased host contact rates. Contrary to our original hypothesis, eastern newt contacts followed a Holling’s Type II functional form, where contacts increased linearly at typical eastern newt densities (e.g., 2–8 individuals/m^2^) but became saturated at simulated high densities (e.g., >50 newts/m^2^). Thus, density-independent transmission might be expected at high newt densities, which could occur during breeding, and is a common attribute of sexually transmitted diseases^53–55^. At lower newt densities, density reduction should reduce *Bsal* transmission. Reducing density from 8 to 2 newts per m^2^ reduced per capita contact rates by 5–15X. Thus, decreasing host density or increasing habitat complexity might be viable disease management options for reducing *Bsal* transmission, although population impacts might depend on host tolerance to *Bsal* infection. We hypothesize that these management strategies will be most effective for amphibian species that have moderate to high tolerance to *Bsal* infection, or if eastern newts are exposed to environmental conditions that negatively impact *Bsal* growth or persistence. It is likely that a combination of disease management strategies will be most effective at controlling *Bsal* invasions in eastern newt populations^56^.

Our study provides evidence that probability of *Bsal* transmission given contact is very high at 14 °C. One contact of one-second duration was sufficient to result in 100% transmission from an infected host at 12–24 days disease progression to a susceptible host. Other contact scenarios (10 and 30-min cohabitation) that we tested and modeled showed nearly identical infection and disease dynamics. Across all treatments, the estimated mortality of susceptible eastern newt hosts due to contact with an infected host was 89%. Hence, high contact rates of eastern newts and efficient transmission of *Bsal* due to direct contact will likely complicate possible disease intervention strategies for this hyper-susceptible host species, as suggested by previous work with fire salamanders^29,57^. Indeed, one caveat that should be considered is that reducing host density or increasing habitat complexity could prolong a *Bsal* outbreak in eastern newt populations^44^. Canessa *et al*. (2018, 2019)^45,57^, suggested that reduction in fire salamander density would need to exceed at least 80% to stop *Bsal* invasion (i.e., reduce R_0_ < 1).

The contact rates that we estimated for eastern newts at densities found in the wild were high. Even at the lowest density tested (2 newts per m^2^), there were on average 93 contacts per day and 1792 contacts per day in the highest newt density, both of which provide ample opportunity for *Bsal* transmission. Although plant structure was provided in the complex habitat treatment, it is possible that natural aquatic systems afford other conditions that facilitate host separation and reduce contact rates. Future research should estimate newt contact rates under more natural conditions. Additionally, our newts were captured during summer when some breeding activity occurs and maintained at 20–22 **°**C, which is typical of summer water temperatures in Tennessee^58^. Hence, contacts may be lower during seasons or in geographic locations with colder temperatures. That said, only 3% of our contacts lasted longer than 20 seconds (i.e., there were few cases of amplexus behavior). Also, ongoing research suggests that eastern newts are more susceptible to *Bsal* at colder temperatures (EDC and MJG, unpubl. data), hence even if contact rates are lower than what we estimated, the probability of *Bsal* transmission given contact may remain high at colder temperatures. We expect that invasion probability of *Bsal* in eastern newt populations will change among seasons^59^, which is the focus of ongoing research.

Our study did not model environmental transmission^60^. *Bsal* transmission can occur via mobile and encysted spores through water and soil^18^. We suspect that addition of these additional transmission pathways will create even faster infection and disease outcomes. Moreover, as infected newt density increases, we anticipate the environmental population of zoospores will grow, and contribute more to epidemiological dynamics^47,61^. Fortunately, it appears that the environmental persistence of *Bsal* zoospores in water with microbes or on soil is probably <1 week^18^. Hence, the contribution of environmental transmission to *Bsal* epidemiology might be a tradeoff between zoospore production by hosts and zoospore degradation in the environment^61^.

Loads of *Bsal* on eastern newt skin increased with disease progression, which was expected, as the infection became systemic and more zoospores and zoosporangia were present. However, interestingly, *Bsal* transmission to susceptible individuals and subsequent development of disease were similar regardless of the infected host disease state, further providing evidence of very efficient transmission between hosts. We suspect eastern newts have few immunological barriers to *Bsal* infection. If skin microbes (e.g., *Pseudomonas*) are proved to provide inhibitory effects on *Bsal*^62^, bioaugmentation of hosts or the environment that can increase the abundance of *Bsal*-inhibiting microbes on the skin might be a viable disease management strategy^63^.

Eleven percent of susceptible hosts (5/45) became infected with *Bsal* yet did not experience disease-induced mortality at 90 days post-exposure and they appeared to clear the infection. These results differ from European fire salamanders, where 100% mortality is observed following exposure to *Bsal*^18,45^. The individuals that survived were across all disease-state and contact-rate treatments, so no relationship between infection intensity and contact was identified. It is possible these individuals had different microbiome attributes that afforded protection^62^, or may have been genetically predisposed to be more immunologically resistant to severe infection. It also is possible that these individuals by chance did not have sufficient host contact to result in a large number of zoospores encysting during transmission. Our results suggest that location of host contact could play a role in *Bsal* transmission. The majority of contacts that we observed were on the head, tail and legs, which also corresponds to the frequent location of *Bsal* lesions^64,65^; (MJG and DLM, unpubl. data). Thus, perhaps contacts for these individuals occurred in locations with lower *Bsal* infection intensity.

Considering the eastern newt’s widespread distribution, high dispersal ability, and high susceptibility, we believe this species will significantly impact *Bsal* epidemiology should the pathogen reach North America. In addition, eastern newts could play a major role in transmitting *Bsal* to other species. The distribution of eastern newts overlaps with some of the highest salamander species richness in North America, and encompasses a global hotspot for Plethodontidae (lungless salamander) diversity. There is growing evidence that several species of lungless salamanders are susceptible to *Bsal* infection and chytridiomycosis^66^. Future research needs to include multiple species interactions and estimate community-level R_0_ under different management scenarios^57^.

Our results suggest a grim outcome for eastern newts and other highly susceptible species if *Bsal* is introduced to North America; thus, actions that reduce the likelihood of introduction are key^57^. The U.S. Fish and Wildlife Service implemented a ban on trade of 201 salamander species due to the risk of *Bsal* invasion^67^. Although this action likely reduced number of *Bsal*-infected salamanders entering the United States via international trade, it did not eliminate the threat. In fact, we now know that frogs can be infected with *Bsal*^18,25^, and anurans dominate (ca. 94%) international amphibian trade in the United States^68^. We recommend that future management actions for *Bsal* include clean trade, where animal health certificates accompany shipments that verify animals are pathogen-free. Currently, no regulations exist in North America requiring clean trade of amphibians, despite three amphibian pathogens (including *Bsal*) listed as notifiable by the World Organization for Animal Health^23,69^. If regulations are enacted, government resources should be allocated to verify compliance and subsidize amphibian trade industries.

## Methods

### Model organism

Adult eastern newts were collected from two wild populations in eastern Tennessee, USA, in Carter County (36.177N, -82.113W) and Knox County (35.847N, -83.872W) in May 2017 and August 2018 under Tennessee Wildlife Resources Agency Science Collection Permit #1504. Following capture, newts were transported by vehicle in <3 hrs to the Johnson Animal Research and Teaching Unit facility at the University of Tennessee and held in large mesocosm tanks at room temperature prior to the experiments. All experiments described herein were approved by the University of Tennessee approved Institutional Animal Care and Use Committee under protocol #2395. All procedures described followed the Association for Assessment and Accreditation of Laboratory Animal Care International Standards.

### Experiment 1: Estimating contact rates among newt densities and habitat complexity

Newts were randomly assigned to one of six circular 1-m^2^ aquatic mesocosms (i.e., experimental units) containing approximately 7 cm of aged, dechlorinated water and maintained at room temperature (20–22 **°**C). Due to space limitations in the laboratory, we tested one host-density treatment (2, 4, or 8 newts) per week. These densities represent a natural range of eastern newt densities in the wild and have been used in other density-focused studies^70–72^. After each week, mesocosms were drained and refilled with aged, dechlorinated water in order to maintain favorable water quality, and we re-randomized newts into mesocosms at a new density to randomly distribute any biases associated with captive duration among experimental effects. For the complex habitat treatment, we repeated the experiment following the same weekly randomization with artificial plants present in the mesocosms. We placed nine artificial plants (ca. 9 cm in height) in a 3 × 3 grid in each mesocosm, with each plant equidistant from each other (ca. 15 cm apart). Newts were fed *ad libitum* by equally dispersing thawed brine shrimp daily throughout each mesocosm. The laboratory was set to 12 hours of light and dark per diel cycle, which is typical daylight duration in Tennessee during summer.

To ensure observers did not influence movements, number of newt contacts per hour was estimated using a surveillance camera (Night Owl, Model: DVR-BBHDA10PB-82-RS; Naples, FL, USA) suspended ca. 170 cm above each mesocosm. Video was recorded for one hour during each of four diel periods (0–0600, 0600–1200, 1200–1800, 1800–2400 hrs), resulting in four hours of video observed per mesocosm per day. We averaged number of newt contacts among the diel periods and days for each mesocosm to obtain a robust estimate of contacts per hour for each host density and plant treatment. One contact was defined as a noticeable contact between two newts, such that one newt responded to contact from the other.

### Experiment 2: Estimating the probability of *Bsal* transmission among disease states and contact frequencies

A separate group of newts were collected from the wild for Experiment 2. Newts were housed individually in 2000-cm^3^ circular containers (diameter = 16.5 cm) with ca. 300 mL of aged, dechlorinated water and a PVC cover object. Because co-infection with *B. dendrobatidis* (*Bd*) could influence *Bsal* infection dynamics^33^, we heat-treated eastern newts upon arrival to the laboratory in an environmental chamber held at 30 **°**C for 9 days^73^. We did not test the newts for *Bd* infection prior to heat treatment, because prior exposure to *Bd* does not seem to connote acquired immunity to *Bsal*^33^. After heat treatment, we decreased temperature over four days to 14 **°**C and held them at that temperature for four additional days before starting the experiment. We verified that all newts were *Bd*-negative using quantitative PCR (protocol discussed below) prior to beginning the experiment. Every three days, we changed housing containers, cover objects and water, and fed newts frozen bloodworms at 2% of their initial body mass.

After the heat treatment, newts were randomly assigned to two groups of either infected or susceptible hosts. Infected hosts (*n* = 15) were exposed to a standardized quantity of *Bsal* zoospores (5 × 10^6^ zoospores/mL) in a 10-mL water bath for 24 hours. We cultured *Bsal* on TgHL plates and harvested them by flooding each plate with a total of 6 mL of autoclaved dechlorinated water and filtering the suspended zoospores through a 20-um filter. Zoospores were enumerated by hemocytometry and verified by flow cytometry. After 12, 18 and 24 days post-exposure (i.e., referred to as disease states in this study), one infected newt was exposed to a susceptible newt through one of three contact frequency treatments: one-second forced contact, 10-min cohabitation, and 30-min cohabitation (*n* = 5 susceptible newts per treatment combination). The one-second contact involved ventrum (infected) to ventrum (susceptible) touching for <1 second then animal separation. For cohabitation, susceptible and infected individuals were co-housed in a 2000-cm^3^ circular container with ca. 300 mL of aged, dechlorinated water for the specified duration. To relate cohabitation treatments to contacts, we recorded and enumerated contacts in each container. The average number of contacts in the 10- and 30-min cohabitation treatments were 19 + 2 and 41 + 6 (s.e.m.), respectively. Location of newt contacts also was recorded for five major body regions: head, tail, legs, dorsum and ventrum.

Following the contact-exposure treatments, susceptible newts were housed in individual containers (same size as above) in environmental chambers at 14 **°**C, newts were inspected daily for signs of *Bsal* chytridiomycosis, and animals euthanized using benzocaine hydrochloride (100 mg/L) at humane endpoints of disease progression. To test for *Bsal* infection, we swabbed infected and susceptible newts every six days following the standardized protocol for *Bd*^74^. Genomic DNA was extracted from each swab using Qiagen DNeasy Blood and Tissue kits (Qiagen, Hilden, Germany), and *Bsal* presence and load on each swab estimated using qPCR methods similar to those described in Blooi *et al*. ^75^. All qPCR reactions were amplified using an Applied Biosystems Quantstudio 6 Flex qPCR instrument. Each swab sample was run in duplicate and considered positive if both replicates amplified within 50 cycles. We confirmed *Bsal* infection on a subset of animals representing each experiment by performing histological examinations of epidermal tissues. The experiment ended at 90 days post-exposure to an infected newt, which is sufficient duration for *Bsal* chytridiomycosis to develop in eastern newts^16^.

### Statistical analyses

For experiment one, we used two-way analysis-of-variance^76^ and Tukey-Kramer post-hoc tests^77^ to test for differences in contact rates among density and habitat complexity treatments. For experiment two, we used Kaplan-Meier survival analyses to compare median survival duration among disease-state and contact-frequency treatments^78^. Because the same infected individuals were used for each subsequent disease-state treatment, we used a linear mixed-effects model^79^ to compare *Bsal* loads in these newts among disease-state treatments (12, 18 and 24-days post-exposure). Post-hoc comparisons of *Bsal* loads among disease states were performed using Tukey contrasts^80^.

We used a hierarchical Bayesian model to estimate the proportion of contacts between newts to be expected across five body regions (head, tail, ventrum, dorsum, and legs) during cohabitation in the second experiment^81^. This approach allowed us to model the contact proportions by body area as a multinomial distribution with parameter values unique to each cohabitation replicate. We modeled each replicate as a multinomial distribution, and parameters were drawn from a Dirichlet distribution using a 100,000 step Markov-Chain-Monte-Carlo following a burn-in of 500 steps. The estimated posterior distributions were interpreted as an estimate of the probability of host contact to each body location for the overall population.

### Epidemiological model

Because our data suggested density dependent contact rates that increased linearly at low densities and reached an asymptotic maximum at higher densities, we modeled host transmission given contact using the following Holling’s type II functional form^82^:

$$f(N)=\frac{cN}{K+N}.$$

here, *f*(*N*) represents the per capita newt contacts per hour for a given newt density *N*; *c* is the value of the asymptote as *N* approaches infinity, and *K* is the half-saturation constant (*f*(*K*) = *c*/2). The parameters *c* and *K* were estimated by fitting *f* to contact and transmission data from Experiments 1 and 2 using multistart with constrained optimization in the MATLAB global optimization toolbox where $0\le c\le 100$ and $0\le K\le 18\times {10}^{4}$. Supplementary Table S1 provides estimates for *c* and *K*.

We modeled *Bsal* transmission, as it occurred in Experiment 2, using a system of ordinary differential equations with three compartments: Susceptible *S*, Exposed *E*, and Infected *I* individuals.

$$\begin{array}{rcl}\dot{S} & = & -\beta \left(\frac{c}{K+N}\right)\,{{\rm{\chi }}}_{[0,10]}(t)SI\\ \dot{E} & = & \beta \left(\frac{c}{K+N}\right)\,{{\rm{\chi }}}_{[0,10]}(t)SI-{\rm{\gamma }}E\\ \dot{I} & = & {\rm{\gamma }}E-dI\end{array}$$

Susceptible individuals (*S*) transitioned to the exposed compartment (*E*) if they remained qPCR negative following cohabitation with an infected newt. Exposed individuals were deemed infected (*I*) and capable of *Bsal* transmission after two qPCR positive swabs. We defined the latency period as the estimated number of days between cohabitation and two qPCR positive results (note: swabbing frequency was every six days).

This model includes the transmission rate (i.e., product of the transmission probability given a contact, *β*, with the frequency interaction term S(I/N) and the density dependent contact rate function $f(N)=\frac{cN}{K+N}$), latency rate γ (from exposed to infected), and a disease-induced death rate *d*. The characteristic function χ_[0,10]_ denotes the number of minutes that the susceptible newts were in contact with infected newts, which in this case was the 10-min cohabitation experiment. For additional details on how parameters were estimated, please see the Supplemental Information file.

Using this system of differential equations, we simulated not only infection prevalence, but also the cumulative proportion of mortality in a population of eastern newts over 90 days due solely to contact transmission. Because model simulations for the 10- and 30-min cohabitation treatments were nearly identical, we present the modeling results in Fig. 6 for the 10-min cohabitation data with and without plants present. We estimated the basic reproductive rate (R_0_) of *Bsal* by using equation 6.21 in Anderson and May (1991:127)^42^ and the cumulative proportion of infected newts for each simulation interpolated from Fig. 6 as the asymptotic level. We decided to not model the one-second contact data, because the treatment represented forced human-induced contact. Also, we performed simulations for all infected host disease states (12–18- and 24 days post-exposure), but simulations are presented for the 12-day infected hosts only because outcomes were similar among disease states. The data sets and code for all statistical analyses and modelling are available in Supplementary File 2.

## Supplementary information

Supplementary information.

Supplementary information2
