# Supplementary material for: Host density and habitat structure influence host contact rates and Batrachochytrium salamandrivorans transmission
Source: Sci Rep. 2020 Mar 27;10:5584. doi: 10.1038/s41598-020-62351-x (PMC7101388; doi:10.1038/s41598-020-62351-x)
Supplement: Supplementary file 1 — Supplementary information. [file 41598_2020_62351_MOESM1_ESM.docx]

**Host density and habitat structure influence host contact rates and *Batrachochytrium salamandrivorans* transmission**

**Daniel A. Malagon^1^, Luis A. Melara^2^, Olivia F. Prosper^3,4^, Suzanne Lenhart^4^, E. Davis Carter^1^, J. A. Fordyce^5^, Anna C. Peterson^1^, Debra L. Miller^1,6^, and Matthew J. Gray^1*^**

^1^Center for Wildlife Health, Department of Forestry, Wildlife and Fisheries, University of Tennessee Institute of Agriculture, Knoxville, TN 37996 USA

^2^Department of Mathematics, Shippensburg University, Shippensburg, PA 17257 USA

^3^Department of Mathematics, University of Kentucky, Lexington, KY 40506 USA

^4^Department of Mathematics, University of Tennessee, Knoxville, TN 37996 USA

^5^ Department of Ecology and Evolutionary Biology, University of Tennessee, Knoxville, TN 37996, USA

^6^Department of Biomedical and Diagnostic Sciences, College of Veterinary Medicine, University

of Tennessee, Knoxville, TN 37996 USA

* Corresponding Author: mgray11@utk.edu

**Supplemental Information**

**Expanded Modeling Description**

The data obtained from the contact rate experiment (Experiment 1), along with the rate of contacts measured in the infection experiment (Experiment 2), suggested that the number of contacts per newt was density dependent (see Results), with linear mass-action response at lower densities yet reached saturation after exceeding 100 newts / m^2^. Thus, we chose the following Holling’s Type II functional form for the number of contacts per newt per hour, which increases approximately linearly for small newt densities and approaches an asymptote as the density of newts increases:

$$f\left( N \right)=\frac{cN}{K+N}.$$

Here, $f\left( N \right)$ represents the per capita newt contacts per hour for a given newt density $N$; $c$ is the value of the asymptote as $N$ approaches infinity, and $K$ is the half-saturation constant ($f\left( K \right)=c/2$). This functional form was incorporated into a mathematical model (see below) to describe the transmission of *Bsal* between susceptible and infected newts. The parameters $c$ and $K$ were estimated by fitting $f$ to data from the experiments using multistart with constrained optimization in the MATLAB global optimization toolbox, and the constraints $0 \leq c\leq100$and $0 \leq K\leq18\times{10}^{4}$. In particular, we determined values for $c$ and $K$ that minimized the sum-of-squared errors between the data and the function $f$ at four newt densities. The first three data points were obtained from Experiment 1 and included the number of contacts per newt per hour when there were 2, 4, and 8 newts per m^2^. The fourth data point was estimated using the contact rate estimated from Experiment 2. In Experiment 2, the density of newts was 2 newts per 0.015 m^2^. This newt density resulted in 112 contacts per newt per hour in the 10-min cohabitation treatment. Therefore, to achieve the same newt density in the 1-m^2^ pool used in Experiment 1 would require $\frac{2 \text{newts}}{0.015 \text{m}^{2}}\times1 \text{m}^{2}\approx133 \text{newts}$. Supplementary Table S1 provides estimates for $c$ and $K$.

We modeled *Bsal* transmission, as it occurred in Experiment 2, using a system of ordinary differential equations with three compartments: Susceptible *S*, Exposed *E*, and Infected *I* individuals. Here, a newt is called ‘infected’ if it tested qPCR positive twice for *Bsal* when swabbed. A susceptible newt became ‘exposed’ if it contacted an infected newt during the experiment and transmission of *Bsal* was successful, but the newt was not yet qPCR positive.


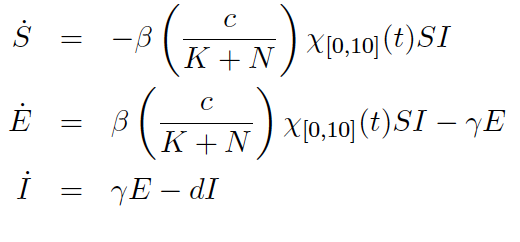


This model includes the transmission rate (the product of the transmission probability given a contact, $\beta$, with the contact rate function $f\left( N \right)=\frac{cN}{K+N}.$), latency rate γ (from exposed to infected), and a disease-induced death rate $d$. The characteristic function χ__[0.,10]_ denotes the number of minutes that the susceptible newts were in contact with infected newts, which in this case was the 10-min cohabitation experiment. We estimated the death rate $d$, using the approximate percentage (20%) of newts that died from the infection in 10 days. The rate at which newts leave compartment *I* due to disease-induced death is given by $\frac{dI}{dt}=-\mu I$. Solving this equation over an interval of 10 days yields the following: $I(10)/I\left( 0 \right)= e^{-10\mu}$. Furthermore, $\frac{I\left( 10 \right)}{I\left( 0 \right)}= 1-0.2=0.8$ is the proportion of infected newts that have survived to day 10, hence $\mu=-\frac{\ln\left( 0.8 \right)}{10}\approx0.0223$ days^-1^. This result implies that the average time to death once infected is $\frac{1}{\mu}\approx45$days.

To estimate the rate γ and probability *β,* the model-simulated cumulative proportion of newts infected at time *t,* given by $C\left( t \right)=\frac{\int_{0}^{T} \gamma E(t)dt}{S(0)}$, was fitted to the data for the cumulative proportion of infected newts obtained from the 10 and 30-minute cohabitation experiments. This fitting was accomplished by creating a vector of values for $0 \leq\gamma\leq2$ and $0 \leq\beta\leq0.98$, solving the SEI model for all possible pairs $\left( \gamma,\beta\right)$ over a period of 90 days (the duration of the experiment), computing the sum of squared differences between $C\left( t \right)$ and the experimental data, and selecting the pair of values with the smallest error as the best fit parameter values. Using this model, we simulated not only infection prevalence, but also the cumulative proportion of mortality in a population of newts over 90 days due solely to contact transmission. Because model simulations for the 10 and 30-minute cohabitation treatments were nearly identical, we present the modeling for the 10-min cohabitation data with and without plants. We decided to not model the 1-sec contact data, because the treatment represented human-induced contact. Also, estimates are provided in Supplementary Table S1 for the different disease states (12- 18- and 24 days post-exposure), but simulations are presented for the 12-day results only because outcomes were similar. Note that *c* and *K* were estimated from the first experiment and did not change with changes in disease state.

**Supplementary Table S1**. Estimates for the model parameters and their sum or squared residuals (SSR) are provided for the different disease states, contact durations, and habitat complexities (with or without plants).
